# Incidence of Malignancy in Children After Cardiac Catheterization Within the First 8 Years of Life Between 1999 and 2013—A Single-Center Experience

**DOI:** 10.3390/jcm15093258

**Published:** 2026-04-24

**Authors:** Heiko Stern, Angela Kretschmer, Alfred Hager, Peter Ewert, Christian Meierhofer

**Affiliations:** 1 Congenital Heart Disease and Pediatric Cardiology, TUM University Hospital, German Heart Center, Technical University of Munich, 80636 Munich, Germany; hager@dhm.mhn.de (A.H.); ewert@dhm.mhn.de (P.E.); 2 Radiation Protection Unit, TUM University Hospital, German Heart Center, Technical University of Munich, 80636 Munich, Germany; kretschmera@dhm.mhn.de

**Keywords:** cardiac catheterization, cancer, radiation dose, children

## Abstract

**Background/Objectives**: Children with congenital heart disease are exposed to ionizing radiation, which may induce cancer. This study aimed to reassess cancer risk after cardiac catheterization (CC) between 1999 and 2013, with follow-up until 15 years of age, cancer diagnosis, or death. **Methods:** We studied 2762 children who underwent at least one CC before eight years of age between 1999 and 2013. Cancer diagnoses were obtained from the German Childhood Cancer Registry. For patients with tumors and 60 randomly selected control patients, cumulative effective radiation doses (Deff) were calculated. **Results:** During 344.80 person-years of follow-up, ten patients developed cancer, whereas 5.3 cases were expected (standardized incidence ratio [SIR] 1.88; 95% CI 0.90–3.46; *p* = 0.0449). Eight tumors occurred in patients who underwent CC during the first year of life, compared with 3.5 expected (SIR 2.26; 95% CI 0.98–4.46; *p* = 0.0282). Patients with cancer had a median of 2.0 (1–11) CCs and a median D_eff of 14.6 mSv (2.4–94.3) compared with 1.0 (1–10) CCs and 9.7 mSv (0.7–171.5) in controls. Neither parameter differed significantly. No specific malignancy was predominant. **Conclusion:** Cardiac catheterization early in life remains associated with an increased cancer risk; however, compared with our previously published 1980–1998 cohort, a reduction in risk was observed.

## 1. Introduction

### 1.1. Background

Congenital heart disease (CHD) is the most common birth defect, occurring in 0.7% of live births [1]. The mortality of children born with CHD has decreased over the past 20 years, resulting in improved survival and longer life expectancy [2].

The median age for low-dose ionizing radiation (LDIR) during diagnostic and interventional procedures in this patient population is decreasing, and these children have a higher likelihood of undergoing multiple LDIR procedures [3,4].

Approximately 95% of the total collective effective radiation dose is attributable to only three procedures: diagnostic catheter, interventional catheter, and computer tomography (CT) [5]. Although radiation doses associated with cardiac catheterization have decreased substantially in recent years despite increasing procedural complexity, children continue to be exposed to ionizing radiation early in life [6]. Cumulative effective doses vary over a wide range [6,7,8], with some patients receiving more than 100 mSv [6] or even 250 mSv, as reported in Sydney, Australia, between 2002 and 2014 [9].

Clinical studies investigating the incidence of cancer in patients with congenital heart defects have yielded conflicting results [10,11,12]. Some recent studies report an increased incidence of tumors [12], while others do not find a higher incidence compared with controls [10,11]. However, the studies differ considerably with respect to the age of the included patients [12], the inclusion of transplanted patients [11], and the types of tumors analyzed [10]. Radiation exposure is generally higher for interventional catheterizations than for diagnostic procedures [13,14].

Such exposure can lead to stochastic biological effects on genes, cells, and DNA, including inflammatory responses [15], alterations in DNA methylation [16], and significant genetic and epigenetic changes [17], resulting in procarcinogenetic effects [18]. Although only about 1% of all cancers occur in children under 18 years of age, approximately 69% are leukemias, lymphomas, and central nervous system tumors [19]. Children who undergo cardiac catheterization at a young age are particularly vulnerable due to higher tissue radiosensitivity and longer life expectancy, which together increase the risk of cancer development [20]. Consequently, the lifetime attributable cancer risk (LAR) associated with radiation exposure decreases with increasing age at exposure [21], with the highest LAR observed in children less than one year of age [5,13]. Most radiation-related cancers in children develop after a latency period of 2–7 years [22,23].

### 1.2. Objectives

This study aimed to assess the risk of cancer in children who underwent at least one cardiac catheterization within the first eight years of life between 1999 and 2013, with follow-up until the age of 15 years, cancer diagnosis, or death. Our previous study, published in 2020, demonstrated a 4.4-fold increased cancer risk in children who underwent at least one cardiac catheterization during the first year of life between 1980 and 1998. The vast majority of cancers developed after a latency period of 2–7 years [22]. We aimed to compare our most recent results with our first study results and investigate whether the effect of cardiac catheterization has changed over time.

## 2. Methods

### 2.1. Study Design

This was a retrospective, observational, single-center study conducted at a tertiary center for pediatric cardiology in Munich.

### 2.2. Patients

The study included 2762 children who underwent at least one cardiac catheterization at the German Heart Center in Munich before the age of eight between 1 January 1999 and 31 December 2013. Of these patients, 1688 received their first cardiac catheterization during the first year of life, and 1074 between the first and eighth year. Patients were required to be residents of Germany. Identity data were encrypted and stochastically matched with data from the German Childhood Cancer Registry (GCCR) to identify subsequent cases of childhood or adolescent cancer.

The following patient data were obtained from either the clinical database or the GCCR: sex, date of birth, date of and age at cardiac catheterizations, dose–area products during catheterizations, death before the age of 15 due to cardiac causes, date and diagnosis of cancer, and preexisting genetic disorders among children who developed tumors.

The study protocol was reviewed and approved by the Ethics Committee of the Technical University of Munich (Project No. 255/14).

### 2.3. Data Sources

Patient data—excluding data on cancer incidence—for all 2765 children who met the inclusion criteria were obtained from our institution’s clinical database. The follow-up period began with the first cardiac catheterization. The end of follow-up was defined as 31 December 2020, the 15th birthday (for children followed until 2008) or 18th birthday (for children followed since 2009), cancer diagnosis, or death due to cardiac disease.

A nested case–control study was subsequently performed, including the identified cases and a control group of 60 patients who had not developed a tumor. The control group was constructed to ensure that the same relative proportion of control patients underwent their first cardiac catheterization before and after the first year of life as the tumor patients. Specifically, 80% of control patients underwent catheterization within the first year of life, and 20% thereafter. Within these two strata (before and after the first year of life), control patients were randomly assigned. For each case and control patient, individual radiation doses were calculated. Only dose calculations from catheterizations performed prior to the diagnosis of the tumor were included.

### 2.4. Estimation of Effective Doses

To analyze the potential relationship between individual radiation exposure and tumor incidence, effective doses (*D_eff_*) were used.

Since calculating the effective dose via individual organ doses (e.g., using the Monte Carlo method) is difficult in practice, it is generally acceptable to estimate the effective dose using certain dose quantities, such as the dose–area product (DAP), combined with appropriate conversion factors. In this study, the DAPs recorded during the catheterization procedures were used.

The effective dose was calculated by first multiplying the recorded dose–area product per plane (*DAP_plane_*) by the corresponding conversion factor $f_{\mathrm{plane}}$, and then summing the resulting individual effective doses to obtain the total effective dose:

$$D_{\mathrm{eff}}=\sum ({\mathrm{DAP}}_{\mathrm{plane}}\times f_{\mathrm{plane}})$$

Dose–area products per plane correspond to the individually recorded DAPs for both planes, anterior–posterior (AP) and lateral (LAT), if both were used during the catheterization.

The conversion factors account for the sensitivity of specific tissues to radiation, considering irradiation geometry, exposure parameters, and tissue-weighting factors.

For the present study, age-dependent effective dose conversion factors for pediatric interventional cardiology were applied according to Karambatsakidou et al. [24] (Table 1).

### 2.5. Statistics

Incidence rates by year, age, and sex were compared using the same registry extract that had been employed for the encrypted stochastic matching. Standardized incidence ratios (SIRs) were calculated based on the observed number of cases and the person-years from the catheterized cohort. SIRs are presented with two-sided exact 95% confidence intervals, as well as a one-sided *p*-value for SIR > 1. The cumulative incidence by age, along with its 95% confidence interval, is shown in the figures.

A multiple linear regression analysis was used to compare the number of cardiac catheterizations and cumulative effective doses between patients who developed cancer and control patients who underwent cardiac catheterization without developing cancer during follow-up. For descriptive statistics, median values with minimum and maximum ranges are reported.

## 3. Results

### 3.1. Patients Included

The catheter database included 3201 children who underwent at least one cardiac catheterization at our institution between January 1999 until December 2013. Of these, 439 children did not reside in Germany and were therefore excluded from the study. Additionally, 181 patients died due to their heart disease and, consequently, their observational person-years were adjusted. As a result, a total of 2762 patients (1389 male) were included, who underwent a total of 4977 procedures. Under these conditions, a total of 34,480.0 person-years could be analyzed. When restricting the analysis to catheterizations performed during the first year of life, 1693 children were exposed, contributing 21,673.7 person-years at risk.

### 3.2. Cancer Incidence

An increased cancer risk was observed in children who underwent at least one cardiac catheterization between 1999 and 2013, compared with the expected cancer risk in the German general population according to the GCCR. Fourteen tumor cases were identified, but four were excluded from the analysis: one patient developed two neoplasms, of which the second was excluded, and three patients were diagnosed with cancer before their first cardiac catheterization. Detailed information on the remaining 10 cases is provided in Table 2. Three of these ten patients also had known chromosomal abnormalities. None of the tumor patients had any comorbidity associated with an increased tumor risk. None of the tumor patients has died to date.

**Figure 1:**
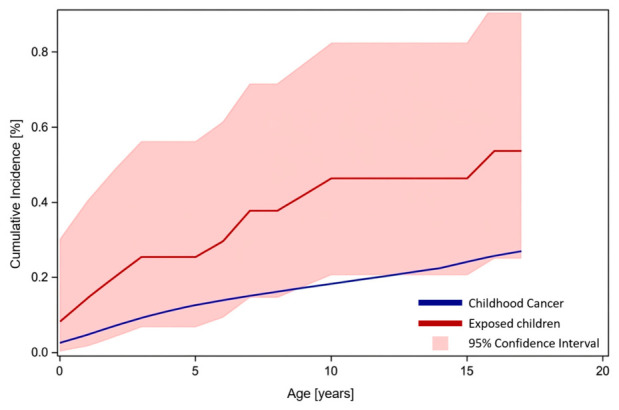
Cumulative incidence of childhood cancer in the general German population (blue line), as provided by GCCR. Cumulative cancer incidence in 2762 children who underwent at least one cardiac catheterization in the first 8 years of life between 1998 and 2013 (red line), with a 95% confidence interval (red shaded area).

Nine children (four female, five male) had developed cancer after catheterization by the age of 10, with one additional case occurring at age 15. The expected number of cases until ages 14/17, based on overall incidence and person-years in the catheterized cohort, was 5.3 (SIR 1.88; 95% CI 0.90–3.46; *p* = 0.0449) (Figure 1).

**Figure 2:**
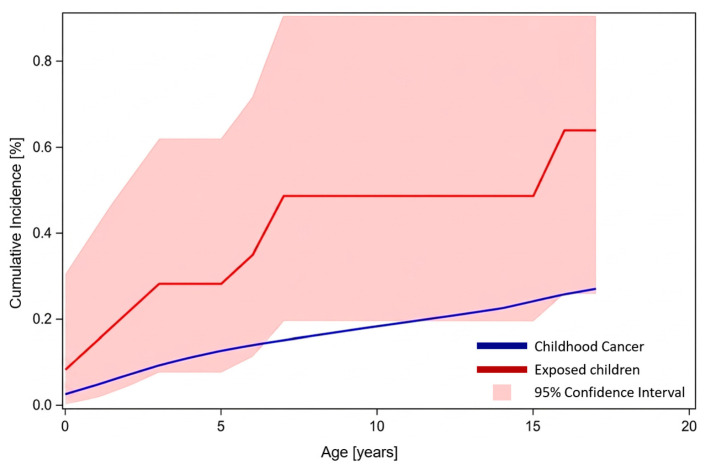
Cumulative incidence of childhood cancer in the general German population (blue line), as provided by GCCR. Cumulative cancer incidence in 1688 children who underwent at least one cardiac catheterization in their first year of life between 1999 and 2013 (red line), with a 95% confidence interval (red shaded area).

Eight of the ten tumors occurred in patients who received their first cardiac catheterization during the first year of life, whereas 3.5 cases were expected (SIR 2.26; 95% CI 0.98–4.46; *p* = 0.0282) (Figure 2). Both SIRs were statistically significantly above 1.0.

No specific tumor type was particularly frequent, and no sex preference was observed. Additional analyses by tumor type were performed, but the numbers were too small to reach statistical significance.

### 3.3. Nested Case–Control Study Including Radiation Burden

**Figure 3:**
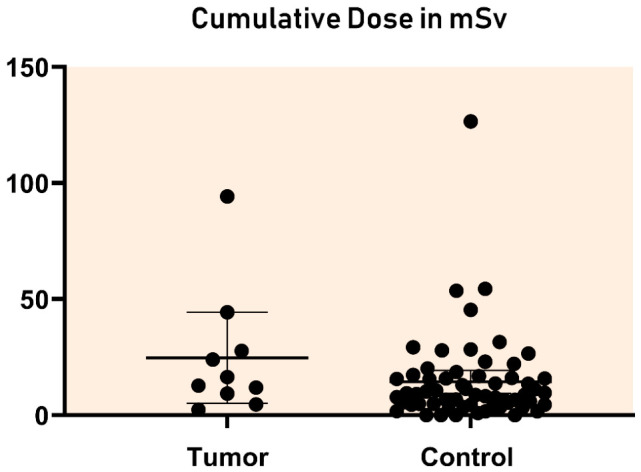
Cumulative effective doses in the children who presented with a tumor and 60 randomly selected control patients.

In the ten patients who developed tumors, a median of 2.0 (range 1–10) cardiac catheterizations were performed, with a median cumulative effective dose of 14.6 mSv (range 2.4–94.3 mSv). In the control group of 60 randomly selected patients, a median of 1.0 (range 1–10) catheterizations were performed, with a median cumulative effective dose of 9.7 mSv (range 0.7–171.5 mSv). Neither the number of catheterizations (*p* = 0.27) nor the cumulative radiation dose (*p* = 0.37) differed significantly between tumor patients and controls (Figure 3).

Focusing on children who underwent at least one cardiac catheterization during their first year of life, patients who developed tumors had a median of 3.0 (range 1–11) procedures, with a median cumulative effective dose of 20.3 mSv (range 9.3–94.3 mSv). The control group had a median of 1.0 (range 1–10) catheterizations (*p* = 0.15), with a median cumulative radiation dose of 9.0 mSv (range 0.7–171.5 mSv; *p* = 0.07). For one patient who developed cancer, the exact cumulative dose could not be calculated because the dose–area product for one of the three procedures was missing. Therefore, the cumulative dose from the remaining two procedures was included in the analysis and presented in Figure 3.

## 4. Discussion

The main finding of this study is a persistently increased cancer risk for children with CHD who undergo cardiac catheterization within the first eight years of life. This effect is even more pronounced when the catheterization is performed during the first year of life. No specific tumor type was found to be predominant.

A major strength of this study is the availability of information on cardiovascular mortality within the study cohort. CHD is still associated with considerable mortality [4]. Therefore, knowledge of cardiovascular mortality is important to determine the exact number of patient-years in follow-up, which affects the person-years at risk and, consequently, the expected number of cancer cases [17].

Our study cohort included children with a wide range of ages, weights, and heights. Therefore, an age-based model was used to calculate effective radiation doses [20], taking into account the higher vulnerability of younger children to ionizing radiation.

The median cumulative effective dose did not differ significantly between children who developed cancer and controls, although the median number of procedures and cumulative radiation dose were higher in the cancer group (2.0 vs. 1.0 catheterizations, 14.6 vs. 9.7 mSv). This suggests that the occurrence of cancer is a stochastic event and is not directly related to the applied radiation dose. Children with complex heart disease are more likely to undergo multiple low-dose diagnostic and interventional procedures [23] resulting in higher cumulative effective doses after multiple catheterizations and an excess related cancer risk [14]. This may be explained by the procarcinogenic effects of ionizing radiation [18] although protective adaptive responses to low-dose ionizing radiation have also been observed [25].

Our finding that eight out of ten patients who developed a tumor received their first catheterization in first year of life supports previous reports that early radiation exposure, particularly during the first year of life, is associated with a higher relative cancer risk [5,8,13,21,26]. An increased risk was also observed in older age groups (18–64 years) among patients with congenital heart defects [12]. However, other studies—which included fewer children under 1 year of age—did not find an increased tumor risk [10,11]

In comparison with our previous study in children with congenital heart defects undergoing cardiac catheterization between 1980 and 1998 [22], the tumor incidence decreased from an SIR of 4.4 to 1.88 in the present study. A major reason for this decline is the absence of patients with trisomy 21 in the current study. In the previous study, 20% of the tumor patients had trisomy 21, which is known to be associated with an increased risk of leukemia [15]. The number of patients with congenital heart defects and trisomy 21 treated at our clinic has significantly decreased since 2000. In the first study involving cardiac catheterization procedures between 1980 and 1998, a catheterization system was used in the first decade that had not been designed for children. The system did not have pulsed fluoroscopy; the frame rate ranged between 50 and 100 frames per second, and for small children under 10 kg, no anti-scatter grid was used, as far as could be determined from the old documentation. All of these parameters changed in the subsequent study involving catheterization procedures between 1999 and 2013 and contributed to lower radiation doses and, consequently, a reduced incidence of cancer. As a result, the tumor group in the first study had a median radiation dose of 43 (0.8–242) mSv, whereas in the current study, the tumor group had a median radiation dose of only 20.3 (9.3–94.3) mSv. Accordingly, the median radiation dose for the control group in the first study was 29 (2–750) mSv, compared to only 9 (0.7–171.5) mSv in the current study.

In general, the decrease in ionizing radiation exposure in recent years are attributable to newer equipment, technical improvement and adaptions, increased awareness of radiation protection, use of radiation reporting systems and better protocols [6,23,27]. However, the number of diagnostic and interventional procedures has also increased [3,4]. Interventional procedures, in particular, deliver on average 23–37% higher radiation doses than diagnostic procedures [13,14].

Potential non-radiation causes of childhood cancer beyond radiation like genetic disorders could not be considered in our evaluation. The finding that three of ten cancer cases were nephroblastoma or hepatoblastoma-tumors known to have low radiosensitivity—suggests that genetic predisposition may have played a role rather than cumulative radiation dose. Refs. [28,29] An increased cancer risk for patients with congenital heart defects is not known but cannot be excluded given the complex etiology of congenital heart defects. It was also not possible to account for radiation exposure from other interventions, such as CT scans; however, CT scans were rarely performed at our institution between 1998 and 2013. Several studies suggest that trisomy 7 is a common finding in various cancer tissues [30,31,32,33]. However, there is no evidence that trisomy 7 itself increases cancer risk, and it is also observed in non-neoplastic cells [34]. Chromosomal abnormalities and DNA alterations can have procarcinogenic effects [18]. Microdeletions 22q11 and 4q34.1 are not reported to increase cancer risk [35].

### 4.1. Limitations

This study focused on radiation exposure during cardiac catheterization within the first eight years of life and the associated radiation burden. Radiation exposure from other procedures could not be considered. Additionally, non-medical risk factors for cancer development, such as environmental exposures or lifestyle factors, were not taken into account. Other confounding factors, such as the lack of detailed genetic analyses that could represent potential risk factors for tumor development, could not be assessed.

Age-related weight changes from birth to eight years result in a wide range of effective doses, reflecting differences in physical exposure conditions. These variations may not be fully accounted for by the age-based dose adjustments used, as the actual radiation burden is not precisely known.

Furthermore, as a single-center study, there may be potential selection bias in the patient population. The patient number is valid for a single center study but relatively small compared to larger multicenter reports. The absolute number of patients who developed cancer was small, which reduces statistical power. Nevertheless, the observed increase in tumor incidence was statistically significant.

### 4.2. Conclusions

Even in the last two decades, children with CHD exposed to ionizing radiation early in life remain at increased risk of cancer. However, compared with our previous study of catheterizations between 1980 and 1998 [19], we observed approximately a halving of cancer incidence.

## Figures and Tables

**Table 1:** Effective dose conversion factors for different ages, posterior–anterior (PA) and lateral (LAT) projections.

Age (Years)	0–0.50	0.51–2.5	2.51–7.50	7.51–12.5	12.51–18.0	
**F PA**	3.65	1.80	0.94	0.62	0.33	mSV/Gy*cm^2^
**F LAT**	3.74	1.97	0.98	0.66	0.34	mSV/Gy*cm^2^

**Table 2:** Information about 10 patients who developed a tumor. Indicated are sex, type of cancer, age at first cardiac catheter, age at tumor diagnosis and genetic disorder. NHL = Non-Hodgkin Lymphoma, NOS = not otherwise specified.

Patient No	Sex	Type of Cancer	Age at First Cardiac Catheter	Number of Catherizations	Age at Diagnosis (Years)	Genetic Disorder
1	M	Precursor cell leukemia	5.4 Months	3	3	-
2	M	Myeloid Leukemia	1.3 Months	1	0	-
3	M	Hodgkin Lymphoma	3.3 Months	1	15	-
4	M	NHL, NOS	4 Days	1	7	Micro- deletion 4q34.1
5	M	Choroid Plexus Tumor	4 Days	8	7	Micro- deletion 22q11
6	W	Astrocytoma	13 Days	2	6	-
7	W	Nephroblastoma	6 Days	1	2	-
8	W	Nephroblastoma	3.5 Years	10	9	-
9	W	Hepatoblastoma	24 Days	1	1	Homozygote MTHFR- Mutation. Free Trisomy 7, Body Dysmorphic Disorder
10	W	Osteosarcoma	5.2 Years	1	10	-

## Data Availability

The data presented in this study are available on request from the corresponding authors due to restricted patient data policy in our institution.
